# Project SCOPE: a National Training Initiative to improve capacity of providers to support children impacted by prenatal opioid exposure using the ECHO model™

**DOI:** 10.3389/fpubh.2024.1478039

**Published:** 2024-11-18

**Authors:** Stephanie Weber, Canyon Hardesty, Andrea Witwer, Ethan Dahl, Elsie Bush, Jessy Thomas, Tai Baker, Ally Dudley, Eric J. Moody

**Affiliations:** ^1^Division of Developmental and Behavioral Pediatrics, University of Cincinnati College of Medicine, Cincinnati, OH, United States; ^2^Division of Developmental and Behavioral Pediatrics, Cincinnati Children's Hospital Medical Center, Cincinnati, OH, United States; ^3^The Wyoming Institute for Disabilities, University of Wyoming, Laramie, WY, United States; ^4^The Nisonger Center at the Ohio State University, Columbus, OH, United States; ^5^Department of Psychiatry and Behavioral Heath, The Ohio State University, Columbus, OH, United States

**Keywords:** Neonatal Abstinence Syndrome, Neonatal Opioid Withdrawal Syndrome, early childhood, interprofessional education, ECHO

## Abstract

**Introduction:**

Neonatal abstinence syndrome (NAS) is a complex condition resulting from prenatal substance exposure that has become increasingly prevalent as a result of the opioid epidemic. NAS can lead to long-term developmental challenges. Interdisciplinary teams with experience working with children with disabilities that focus on social determinants of health can be effective at supporting families affected by NAS. Unfortunately, interdisciplinary teams often lack sufficient training, ongoing practice support, and public health policies to support these families. The objective of this project was to determine the feasibility and impact of a National Training Initiative, called Project SCOPE, to improve the capacity of providers to address the needs of children with NAS and their families.

**Methods:**

Fourteen (14) sites were trained to fidelity in the ECHO model and SCOPE curriculum, and then each team implemented this model for at least one, eight to 12 session series between 2019–2022. The reach, impact, satisfaction, and intention to implement the model were assessed from administrative records, pre/post surveys, and post-session evaluations.

**Results:**

SCOPE state teams delivered the curriculum to 9,392 individuals across 33 US states. Surveys from 2,197 individuals were used for analysis. Most participants (84%) had previous training in trauma informed care, but only 53% had any training on the NAS or the opioid crisis' impact on children. Satisfaction with SCOPE sessions was high (96.4%), and there was a statistically significant increase of self-reported knowledge change from pre- to post-SCOPE. Over 94% reported their skills increased because of participation. Over 97% of participants indicated their motivation to work with this population increased from SCOPE participation and that they could successfully apply what they learned. Almost 70% reported they were “very” or “extremely” likely to use their new skills.

**Discussion:**

Project SCOPE is a highly effective and impactful model that can radically improve capacity to support children affected by the opioid epidemic, thereby increasing the capacity of our healthcare system to respond to this epidemic. Moreover, this model can be rapidly deployed and reach a wide geographic region, especially areas that are affected by the opioid crisis and underserved rural communities.

## Project SCOPE: a National Training Initiative to improve capacity of providers to support children impacted by prenatal opioid exposure using the ECHO model™

Substance use disorders (SUD) during pregnancy can lead to Neonatal Abstinence Syndrome (NAS)^1^ in prenatally exposed children (1). Similar to those with developmental delays or disabilities, children with NAS may present with complex physical, mental, and behavioral health and social support needs requiring the collaboration of experienced practitioners, researchers, and leaders across domains of healthcare, public health, early intervention, education, mental and behavioral health, family support, and social services (2, 3, 46). Therefore, caring for and educating children with a history of NAS presents unique challenges given the cross-system collaboration necessary to effectively reduce the public health burden.

Unfortunately, the existing healthcare workforce is insufficient to address the clinical needs of the growing population of infants with NAS or mitigate the public health impact. For example, since 2010, Maternal Opioid-Related Diagnoses (MOUD) almost doubled from 4.6 per 1,000 delivery hospitalizations to 8.2 per 1,000 (4). The increase in opioid use during pregnancy has contributed to an increased U.S. prevalence of NAS from 4.0 in 1,000 births in 2010 to 7.3 in 1,000 births at the peak in 2017 (5). Infants with NAS are at increased risk for a variety of complications shortly after birth, such as tremors, seizures, feeding problems, and they may require pharmacological treatment and/or intensive care admissions (6). Clinical care research has emerged to demonstrate effectiveness of the Eat, Sleep, Console approach to decrease length of hospital stay compared to usual care (48). However, while most infants recover from the immediate withdraw symptoms (7), there is growing literature to suggest that NAS can have effects that persist well into early childhood and adolescence (8–13). For instance, children between the ages of 3 and 8 years with a history of NAS are more likely to be diagnosed with developmental delay or speech impairment and more likely to receive school-based supports (8). Preschool-aged children with a history of NAS have also shown deficits in motor skills and memory ability, poor peer relationships, hyperactivity and short attention span, impulsivity, aggressive behavior, and increased risk of developing Attention-Deficit/Hyperactivity Disorder [ADHD (9, 10, 12–17)].

Given the possible multi-faceted needs of children with NAS and number of systems that may be required to effectively support these children, interdisciplinary team-based approaches are apt to be more effective (18). Moreover, there is growing recognition that the conditions of the environments where people are born, live, learn, work, play, worship, and age affect a wide range of health, functioning, and quality-of-life outcomes and risks (US Department of Health and Human Services). Addressing these social determinants of health [SDOH (19)] requires that a robust interdisciplinary workforce has access to training and continuing professional development on emerging evidence-based SDOH practices.

Indeed, public health approaches [e.g., the 10 essential public health services (20)] call for equitable access for all by building a diverse and skilled workforce, based on innovative, evidence-based programs that ensure strong organizational infrastructure for public health. Therefore, innovative training approaches/models are needed to enhance the capacity of interdisciplinary teams. However, to ensure effectiveness of such programming, the training delivery method should be flexible, collaborative, acceptable, and effective. Such programs should have minimal lead time from conceptualization to implementation to ensure rapid uptake of best-and practices and should easily adapted for use by various communities to meet their own unique needs. One such promising delivery method is the ECHO model™ (21).

## The Extension of Community Healthcare Outcomes model for professional development

The Extension of Community Healthcare Outcomes, or Project ECHO, was developed as a model of professional development (PD) that adheres to the principles of effective PD (21–25). Specifically, content delivered through ECHO is directly relevant to current professional struggles (26, 27) and includes four core features of effective adult learning: (1) delivery of content knowledge, (2) active learning (i.e., hands on work), (3) integration of changes into daily professional life, and (4) continued follow up and support after training to allow for continued adaptation of the skills (24, 27–31).

The Project ECHO model includes (1) short professional development trainings, (2) case-based learning through case-narratives, (3) ongoing co-management and individual coaching and (4) robust program evaluation (21). All of this is conducted over video conferencing, which allows professionals in distinct settings to receive highly relevant PD and receive specific guidance related to their current needs and concerns. This means that interdisciplinary teams are trained to work more effectively with their clients, rather than simply be presented with general and vague information that may not be relevant to their current situation. Participants of ECHO are, therefore, able to be better prepared to respond to emerging public health needs, which improves the capacity and diversity of the workforce that supports children with NAS. Providing ongoing support to those working with children and families impacted by the opioid crisis is consistent with The 10 Essential Public Health Services (20).

The primary goal of the current project was to develop, disseminate, and evaluate the implementation of a model of PD using a core curriculum to meet the immediate and complex needs of the workforce supporting children and families impacted by the opioid crisis, specifically children with a history of NAS. Project SCOPE (Supporting Children impacted by the OPioid Epidemic) aimed to enhance the public health workforce rapidly and effectively to increase capacity for interdisciplinary community providers to address this issue. Project SCOPE was a 3-year National Training Initiative (NTI) intended to build nationwide provider capacity and confidence in applying evidence-based practices. The primary goal was to determine the impact of a large, multi-state implementation of Project SCOPE and its potential to build healthcare workforce capacity to address NAS on a large scale. This project was based on the ECHO model to ensure that it can quickly and effectively meet the needs of a broad range of communities that are impacted by public health issues related to NAS and the opioid crisis.

## Method

### Project SCOPE National Training Initiative

Project SCOPE was a collaboration between three University Centers of Excellence in Developmental Disabilities [UCEDD; i.e., Wyoming Institute on Disabilities (WIND), Nisonger Center, and University of Cincinnati Center for Excellence in Developmental Disabilities (UCCEDD)]. The UCCEDD and Nisonger Center developed the SCOPE curriculum, and WIND leveraged their expertise with the ECHO training model to structure the implementation of this curriculum. The NTI lead team trained 14 state teams to implement the core SCOPE curriculum developed during a 2018–2019 pilot phase. During the spring of 2019, a robust evaluation plan was implemented for the pilot study to determine the feasibility, reach and impact of this model of PD. Four key outcomes were evaluated: (1) network reach and participation, (2) participant satisfaction and relevance to their practice, (3) participant knowledge and skill development, and (4) participant intention to implement new skills. This framework was then replicated for the current NTI project. A base set of evaluation measures created during our pilot phase was used at all sites; although, each site could add additional measures as desired. Note that additional site-specific measures are not reported here. During the fall 2019 to spring 2020, state teams applied to receive training on the ECHO model and core SCOPE curriculum. Each state team was led by a UCEDD and/or Leadership Education in Neurodevelopmental Disabilities (LEND) program. UCEDDs and LENDs are part of a nationwide network of centers that are enabled by the Developmental Disabilities Rights Act (32) to provide training, research, supports, model programs and other supports to address the needs of those with disabilities.

The following states implemented SCOPE as part of this NTI project: Arizona, Colorado, Georgia, Kentucky, Maine, Minnesota, New Hampshire, New York, North Dakota, Ohio, South Dakota, Utah, Vermont, and West Virginia. Maine and New Hampshire served as a joint team for this project. Each state was trained to fidelity in the use of the ECHO model and the SCOPE curriculum. This was done remotely given COVID-19 restrictions. Two-day training sessions were conducted by WIND ECHO Superhub staff (experts in the ECHO model) and content experts on NAS from the Nisonger Center and UCCEDD. The training team included clinical psychologists, public health professionals, occupational therapists, and other professionals with NAS expertise. Each state team then implemented SCOPE and was responsible for recruitment, delivery and evaluation of their state implementation using a standardized evaluation framework developed by the lead sites. Each site adapted the implementation timeline to meet their local needs, which ranged from 8 to 12 weeks and was implemented between 2019 and 2022. Given that ECHO is delivered over Zoom™, the public health emergency due to the COVID-19 pandemic did not impact implementation significantly. However, the use of teleconferencing as a primary means of interaction was still new to some of the NTI's target professionals at this time. All data were collected electronically using REDCap (33) and iECHO (a proprietary administrative reporting tool used by all ECHO implementation sites). Each site received $17,500 for participation in the training and implementation of three SCOPE series.

### Participants

After each state team was trained, they implemented SCOPE locally. Practicing professionals (*N* = 9,392) were recruited by the local state teams through a variety of methods such word-of-mouth, e-mail, and social media. Programs that were targeted for recruitment included state-wide early intervention systems (Individuals with Disabilities Education Act Part C), early childcare and education (e.g., preschool, childcare, and Head Start centers), healthcare professionals serving children birth to age five, and family members. Participants were free to join Project SCOPE sessions at no cost. Each implementation site was free to use an open or closed cohort design. Participants were eligible if they worked with children at risk for NAS, regardless of professional capacity, or were family members of children with NAS. All participants were invited to complete all surveys by the host site, which allowed them to register to participate in the SCOPE sessions.

### Measures

Evaluation of SCOPE focused on the following outcomes: (1) network reach and participation, (2) participant satisfaction and relevance to their practice, (3) participant knowledge and skill development, and (4) participant intention to implement new skills. Data sources included administrative measures, traditional pre-post surveys at the beginning and end of SCOPE participation, and short evaluations after individual ECHO session. For pre-post surveys and session evaluations, a retrospective pre- then post-survey was used to account for reporter bias (34). Specific measures for each of our four outcome areas are below. Note that participation was completely voluntary, and participants were free to skip any question.

#### Network reach and participation

These data came from three sources: administrative data from the iECHO platform, a 25-item demographic form, and a survey of to what degree adverse childhood experiences impact their clients. The iECHO platform is the standard administrative data reporting tool used by all ECHO implementation sites. It captures the name, contact information, and practice location of all attendees. Name and contact information were redacted for this analysis. Zip code was used as a proxy for participant location given that many participants joined from extremely remote locations. Zip codes were converted to county to limit the potential for inadvertent identification of participants in extremely rural or frontier areas. These data were then used to generate heat maps by state and county to understand the reach of SCOPE.

A 25-item demographic form captured information on participant age, race, ethnicity, income, education, job, job setting, years of job experience, years of childcare experience, experience with NAS and trauma related care, and interest in topics related to NAS. This form was internally generated by the research team. The adverse childhood experiences survey is an internally generated 22-item questionnaire asked participants to indicate “Yes” or “No” if they worked with any children who had experienced or were experiencing any of the listed adverse experiences. If a participant responded “Yes” to a particular experience, they were asked to indicate the number of children they were working with who had that experience. Both these instruments were used to characterize the SCOPE participants, as well as the degree to which their clients have experienced adverse experiences using descriptive statistics.

#### Participant satisfaction and relevance to their practice

Participant satisfaction and relevance to their practice was captured using the post-session evaluations and the final post-SCOPE survey at the end of an entire series. Satisfaction with the speakers and topic(s), relevance to their work, and usefulness of the material were captured following each session. The post-SCOPE survey asked questions about an individual's overall learning and experience during their participation, evaluating improvement of one's quality of practice, further demonstrating session relevance.

#### Participant knowledge and skill development

Knowledge and skill development were evaluated using an internally generated opioid knowledge survey (OKS), post-session surveys, and the post-SCOPE survey. The OKS was an objective measure of knowledge of the learning objectives covered as part of SCOPE sessions. Participants completed the OKS before attending any SCOPE sessions and after the SCOPE series had concluded. The first version of the OKS contained 15 multiple-answer and multiple-choice items and scores ranged from 0 to 15, where participants received a point for each correct answer. The OKS was revised in 2021 to add six additional items to further explore knowledge gain. Both versions were scored using a sum score and change from pre- to post- using paired sample *t*-tests.

Post-session surveys identified participants' self-reported knowledge before and after the session, as well as the likelihood of using the knowledge they had gained. Post-SCOPE surveys identified knowledge increase, skills increase, likelihood of continuing to use new knowledge/skills, and a retrospective pre-post measure of knowledge related to learning objectives prior to the session, current level of knowledge, and participant rated level of knowledge needed to be successful in a participant's job. All items were on 5-point Likert scales.

#### Participant intention to implement new skills

The intention to implement new skills was captured in both post-session and post-SCOPE surveys. For post-session surveys, intention to use new skills was measured via a 7-point scale ranging from 1–False to 7–True. After each session, participants were asked to endorse items that framed the strategies and skills as: “I plan to…,” with session strategies and skills listed at the end of each item. Confidence in one's ability to implement new information or skills, plans to use new skills, how often participants predicted using skills, and a timeline to implementation were evaluated in the post-SCOPE survey. Participants also indicated whether they had used specific skills and if they intended to use those skills in the future. Additionally, participants were asked to report whether their quality of practice and motivation had increased due to attending SCOPE sessions.

## Procedure

After each state team was trained, they implemented SCOPE at least once. Prior to implementation, each site executed a licensing agreement with the ECHO Institute to implement ECHO to fidelity. Each SCOPE series ran between eight to 12 sessions. Aligned with the ECHO model, the exact number of sessions was decided upon by the state teams based on local needs. All SCOPE sessions met on a consistent schedule for either 60 or 90 min each at a time convenient to accommodate professional schedules. Sessions followed a specific structure to maximize learning, engagement, and application of best practices. Sessions included introductions and a short didactic presentation by a content expert followed by a case-based learning opportunity.

All evaluations were administered using a REDCap™ (33) database managed by the University of Wyoming. All state lead team sites collected data related to the sessions they conducted. Separate REDCap™ projects were created for each site. Sites were allowed to add additional questions to a predetermined core evaluation; however, no questions were allowed to be removed from the core evaluation. Participants completed measures through a REDCap™ database prior to their first SCOPE session (pre-SCOPE) and following each session attended (post-session) as well as following the entire series (post-SCOPE). Demographic and adverse childhood experiences forms were completed pre-SCOPE. The OKS was collected pre- and post-SCOPE. The post-SCOPE survey was completed after the conclusion of each sites' SCOPE series.

### Analysis

This is a program evaluation of an NTI using the ECHO model to improve workforce capacity. We used descriptive analyses to explore each of the outcomes of interest listed above. Means, standard deviations, percentages, heatmaps, and bar charts were used to describe the distributions and visualize the data. Where appropriate, changes from pre- to post-survey were tested using paired sample *t*-tests (alpha set at 0.05) and descriptive statistics. All analyses were conducted using SPSS version 28.

To examine the reach of SCOPE, we created heatmaps to visualize the location of participants, along with density of participation. We also characterized the number and types of practitioners that were trained through this model, as well as the estimated caseload of children potentially impacted by the training. We then calculated the average satisfaction of all sessions, across all sites. Next, we evaluated the impact of the training on the core knowledge and skills of the providers. Subjective changes in knowledge were measured during pre- and post-SCOPE surveys and surveys following each session. This was captured using a traditional pre-post design, as well as a retrospective pre-post to account for bias. These were then compared to the respondents' knowledge goal (i.e., what level of knowledge was desired). Also, paired sample *t*-tests were used to measure knowledge changes after each session. Note that in the case of the OKS, separate paired sample *t*-tests were used for each version. Finally, we evaluated the providers' intention to implement their newly acquired skills, their use of the strategies or skills, and implementation of the knowledge gained as a part of the SCOPE community. Means, standard deviations, and bar charts were used to characterize these measures.

## Results

### Characteristics of participants

**Figure 1:**
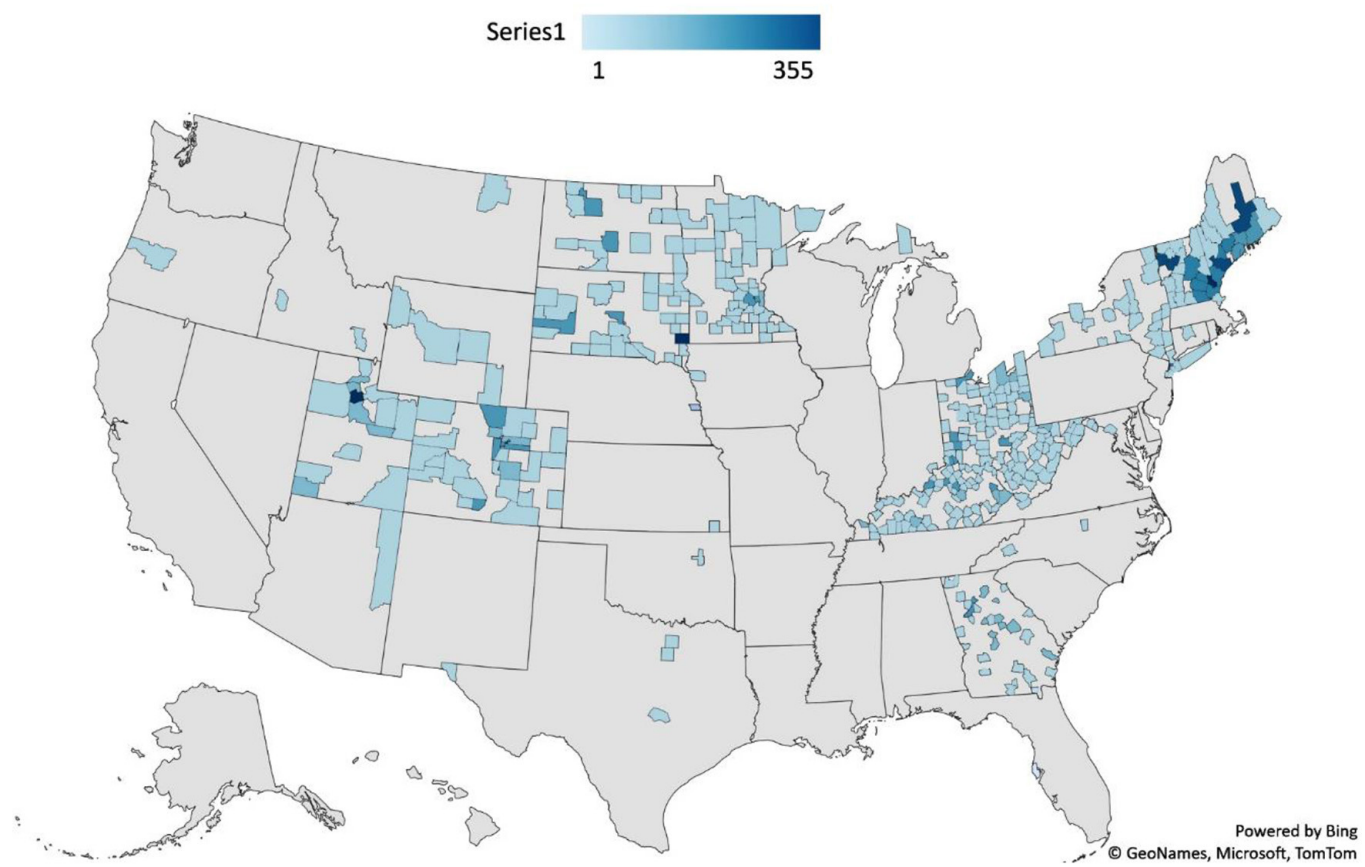
Location of participants in SCOPE by state and county.

Based on administrative data from iECHO, 9,392 individuals participated in at least one ECHO session and came from 33 states: Arizona, California, Colorado, Connecticut, Florida, Georgia, Idaho, Indiana, Iowa, Kansas, Kentucky, Maine, Maryland, Massachusetts, Michigan, Minnesota, Montana, Nebraska, New Hampshire, New York, North Carolina, North Dakota, Ohio, Oklahoma, Oregon, South Dakota, Texas, Utah, Vermont, Virginia, West Virginia, Wisconsin, and Wyoming. Figure 1 shows a heatmap of the US with state and county as the units of analysis. As expected, most participants came from states that had teams participating in this project. However, many participants joined from all over the country. Density of participation varied greatly from state to state. Given that many states are considerably more rural than others, that is not unexpected. However, many states had broad participation from a majority of their counties.

A total of 2,197 individuals provided at least some demographic or professional background information. Each question was analyzed separately to maximize the sample, which varied between 1,968 and 2,172 respondents. The overall sample represented a wide range of backgrounds and professions. Those identifying as women accounted for most participants. Men and those not reporting a binary gender accounted for the minority of participants. Most participants were highly educated with 89.9% possessing a bachelor's, graduate, or professional degree. Most participants identified as White, but there were significant proportions from other racial and ethnic groups. Black or African American and Hispanic/Latino were the largest non-White groups, with all other groups accounting for small percentages. Age was normally distributed, with most participants between 35 and 54 years old. See Table 1 for demographics.

**Table 1:** Demographic characteristics of SCOPE participants.

	***n***	**%**
**Participant location** ^**^		
Colorado	1,153	12.4
Georgia	822	8.8
Kentucky	1,068	11.4
Maine	702	7.5
Minnesota	436	4.6
New Hampshire	872	9.3
New York	572	6.1
North Dakota	199	2.1
Ohio	1,278	13.6
South Dakota	747	8.0
Utah	673	7.2
Vermont	508	5.4
West Virginia	202	2.2
Other	160	1.7
Total	9,392	
**Gender**		
Female	2,052	94.6
Male	111	5.1
Other/not reported	5	0.2
Total	2,168	
**Education level**		
Some High School	1	0.0005
High School Graduate or GED	30	1.4
Some College or Post-High School or 2 Year Degree	189	8.6
College Graduate	832	37.9
Advanced Graduate or Professional Degree	1,120	51.0
Total	2,172	
**Race**		
White	1,781	82.6
Black	163	7.6
Asian	29	1.3
Hispanic or Latino	86	4.0
Prefer to Self-Describe	94	4.4
More than 1	2	0.1
Total	2,155	
**Age**		
< 18	15	.7
18–24	78	3.6
25–34	467	21.3
35–44	570	26.3
45–54	601	27.7
55–64	342	15.8
65+	100	4.6
Total	2,167	

^**^Data come from iECHO system, states implementing SCOPE listed with all other states listed as “other.”

Participants' occupations were diverse. The most commonly reported occupation was “other,” (*n* = 752) although, participants included social workers (*n* = 298), early intervention specialists (*n* = 214), education professionals (*n* = 63), medical professionals (Physicians, developmental behavioral pediatricians, child psychiatrists, nurse practitioners, *n* = 61), legal professional (attorneys, judges, guardian ad litems; *n* = 7). The average years in current professional role was 8.29 years (SD = 8.41), but there was a large range (from 0 to 47 years). The average number of years working with children aged 0–5 was 12.74 years (SD = 10.37), ranging from 0 to 50 years of experience. The current work setting of the participants was predominantly home-based, which is consistent with the age of the children who would be affected by this project. However, there were many participants from other settings such as schools, clinics, and childcare organizations. The participants served in a wide range of systems, ranging from government (e.g., Part C), hospitals and clinics, and childcare programs. However, the majority worked in “Other,” which were not one of the predetermined answer choices (see Table 2).

**Table 2:** Professional experience of SCOPE participants.

	***n***	**%**
**Primary system of profession**		
Part C/Early Intervention	464	21.9
Part B/Special Education	114	5.4
Head Start	157	7.4
Early Head Start	57	2.7
Childcare	153	7.2
Home Visiting	197	9.3
Hospital/Medical Center	230	10.8
Private Practice	54	2.5
Other	696	32.8
**Experience working with children with prenatal opioid exposure**		
Yes	1,365	64.2
No	478	22.5
I Don't Know	282	13.3
**Received any other training related to trauma or trauma-informed care**		
Yes	1,796	83.8
No	347	16.2
**Received any other training on the topic of the opioid crisis and its impact on children**		
Yes	1,132	53.0
No	1,003	47.0

Most participants reported working with children who had been exposed to opioids *in utero*. The average number of children on their caseloads with opioid exposure was 5.6 children, accounting for 141,085 children across all training sites, indicating a substantial impact of opioid exposure which is consistent with the ongoing opioid crisis in the US. Most participants (84%) had received some training in trauma informed care, but only 53% had received training on the opioid crisis and its impact on children, suggesting limited training option on the impact of NAS.

### Satisfaction and relevance

**Figure 2:**
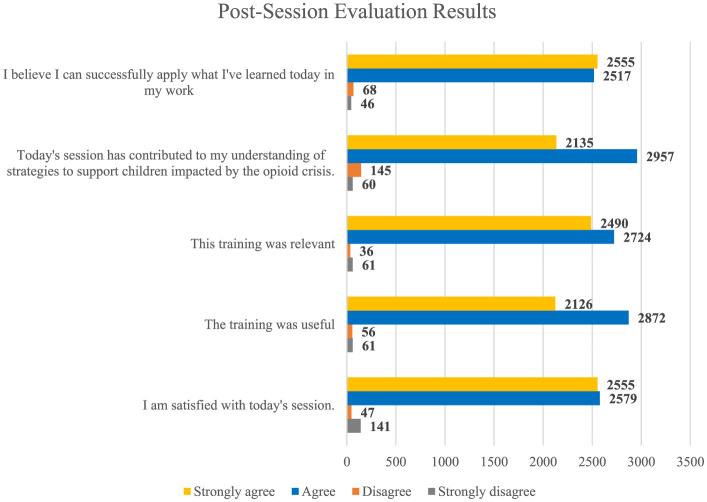
Participant self-reported understanding, satisfaction, relevance, and application after SCOPE.

Satisfaction with SCOPE was high, with 96.4% of participants reporting satisfaction with the sessions and 96.1% endorsed that the sessions contributed to their overall understanding of strategies to support children impacted by NAS. Additionally, in relation to personal relevance, 98.1% of participants felt that the learning topics were relevant and 96.1% of participants reported the weekly sessions contributed to their understanding of the opioid crisis and how it impacts children (see Figure 2). Further, most participants felt attending the sessions helped them connect to other practitioners, and that SCOPE expanded their professional networks.

### Knowledge and skill gains

#### Opioid knowledge survey results

Results for version 1 of the OKS (prior to 2021) showed a statistically significant increase in knowledge from pre (M = 8.50, SD = 1.65) to post (M = 9.02, SD = 1.40); *t*_(114)_ = 3.33, *p* = 0.001. The effect size, measured by Cohen's *d*, was *d* = 0.31, indicating a medium to small effect. Similarly, results for version 2 showed a statistically significant increase in knowledge from pre (M = 13.07, SD = 2.13) to post (M = 14.02, SD = 2.04); *t*_(55)_ = 2.54, *p* = 0.007. The effect size was also medium to small with a Cohen's *d* of 0.34.

#### Post-SCOPE survey results

**Figure 3:**
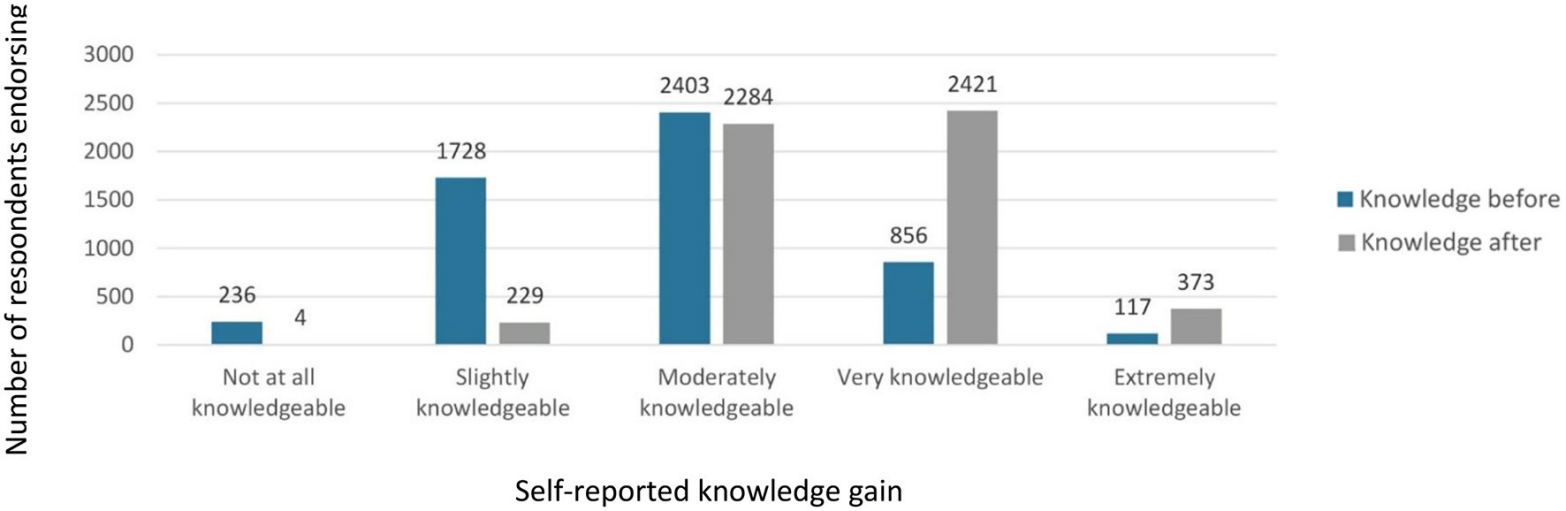
Participant self-reported knowledge before and after completion of SCOPE.

Most participants (96.1%) rated knowledge improvement due to participation in SCOPE sessions, and 94.5% felt that their skills improved at least some. There was a significant increase in self-reported knowledge from before participating in SCOPE (M = 3.32, SD = 1.0) to after participating (M = 4.17, SD = 0.94), *t*_(410)_ = 23.43, *p* < 0.001 (see Figure 3). The effect size was large, with a Cohen's *d* of 1.16. Though, the knowledge level after participating in SCOPE (M = 4.17, SD = 0.94) was not significantly different than the knowledge level participants reported they need to be successful (M = 4.12, SD = 1.07), *t*_(411)_ = 1.17, *p* = 0.24. The effect size was small with a Cohen's *d* of 0.06. Knowledge also significantly increased following each of the sessions. Using a retrospective pre- then post design, respondents reported a significant increase in knowledge from before the training (M = 2.74, SD = 0.74) to after the session (M = 3.51, SD = 0.60), *t*_(1, 560)_ = 54.07, *p* < 0.001. The effect size was large with a Cohen's *d* of 1.37.

### Intention to implement

Ninety-two percent reported their practice quality improved due to participating in Project SCOPE. Further, 97.4% of respondents said that their motivation to work with children impacted by opioids had increased. Importantly, 97.8% of participants, or 5,072 of the 5,186 responses to weekly session surveys, said that they could successfully apply what they learned in their practice. This confidence is confirmed from their actual usage, with 84.0% of respondents indicating that they had used new knowledge from the SCOPE session in their work. Moreover, it seems once trained, participants could rapidly implement what they had learned. When asked the degree of how likely they would use their new knowledge, 93.1% said they were at least moderately likely to use their new knowledge, with 69.0% saying they were “very likely” or “extremely likely” (see Figure 3).

Participants were asked to report on their confidence related to the use of specific strategies or skills taught in the SCOPE sessions and their intent to use these strategies and skills in the future. Fifty-six percent of participants reported having used the strategies at the time of the post-SCOPE survey. Further, 67.0% said that they intended to use one more of their new skills in the future. Moreover, 93.7% of participants said that they were “likely” or “extremely likely” to continue using these skills in the future. Most respondents also indicated that they would likely use these skills in the near future, with 55.7% saying they would use the skills immediately or within a few weeks and another 23.7% indicating they would use their new knowledge within a few months.

## Discussion

As hypothesized, this program evaluation found that SCOPE was effective at rapidly increasing the capacity of the interdisciplinary workforce to serve children and families affected by NAS. Specifically, SCOPE was able to be deployed rapidly through the ECHO model and successfully increase the skills and knowledge of providers. Moreover, SCOPE allowed providers to quickly start using their newly acquired skills and has the potential to impact a large number of children at risk for developmental challenges across a broad geographic range.

One of the most striking findings of this analysis is how effectively this approach was able to quickly reach and significantly impact the NAS workforce. Specifically, participants had a large increase in knowledge over a relatively brief period of training that was at the level they said they would need to be successful. This included both their reported knowledge level as well as their direct knowledge of opioid related information. Moreover, their confidence in their ability to use their new knowledge was rated as extremely high with over half of the sample intending to use their skills within a few weeks. Further, most participants indicated that they were “likely” or “extremely likely” to continue using their new knowledge and skills. This suggests that SCOPE can quickly increase the capacity of the NAS workforce and give professionals skills that are immediately relevant to their current and future caseloads. The speed with which this program can be deployed, and its broad reach make it a valuable tool to train and support professionals managing the developmental challenges of NAS.

Given the complexity of meeting the needs of those with NAS and the multiple professions and systems involved (18), interprofessional development to expand the workforce trained to address this public health problem is critical. By specifically developing a curriculum based on emerging evidence and best practices, our NTI was able to ensure that professionals from a range of disciplines across 14 states received the most up-to-date practice information in SUD and NAS. Overall, this model allowed novel strategies used throughout the network to be implemented broadly, thereby increasing capacity and the infrastructure needed for public health and policy initiatives.

This contrasts with many professional development strategies which are notoriously slow, costly, and do not effectively support adult learners (35). Given this, SCOPE may be a particularly valuable tool to address the major public health implications on children of the opioid crisis. This project adds to the growing body of research that demonstrates that the ECHO model is particularly effective at delivering relevant and timely information to providers that allows them to provide equally effective care as specialists [e.g., (36–38)].

Another striking feature of this project is the broad reach of SCOPE. As can be seen from the heat maps, SCOPE was able to reach an extremely diverse geographic range. This included both urban and rural areas, more compact eastern states and large western states. Participation was clearly centered around states that were part of the NTI, but participants joined from many other states as well. This suggests that professionals are searching out professional development opportunities related to NAS and that the ECHO model can support practitioners who would otherwise not have received the training offered by SCOPE. This, along with the high satisfaction scores suggest that SCOPE is a highly acceptable training model.

It is also noteworthy that participants from many counties were in rural or frontier areas. Traditionally, these areas struggle to meet clinical needs (39) and encounter persistent health care shortages (40). That SCOPE was able to train many providers from rural areas is a particular strength of this model. Indeed, ECHO was originally developed as a tool to support physicians in rural communities (41), and these data further demonstrate the success of ECHO for this purpose. In addition to rural and frontier participants, a large number of providers joined from urban areas as well. This could be due to the immense need in those communities, the ease of use of the ECHO model, or both. This demonstrates that ECHO is able to reach rural and underserved communities that traditionally struggle with access to specialty care.

The estimate of the number of children impacted by SCOPE is also striking. Although this is a self-reported estimate, and not an actual count of impacted children, this still demonstrates that SCOPE can potentially reach an extremely large number of children in need for a relatively modest investment of time. It is also worth noting that the skills professionals gained through SCOPE are applicable well into the future for other children with developmental concerns and/or complex psychosocial histories (e.g., complex traumatic stress). For example, having greater awareness of trauma (a key topic in SCOPE) may lead clinicians to be more attuned to trauma reactions in children who present for non-NAS related problems. Further, that clinicians can use these skills immediately may allow them to maintain their knowledge more effectively and make it part of their ongoing practice. Indeed, previous research suggest that to be effective, professional development needs to have opportunities to practice the skills (42). This conforms to adult learning theory (43) and is a founding principle of the ECHO model.

Results indicate that many participants work directly with children at-risk for NAS and reported increased confidence in implementing strategies, increased connection to the community of practice, high intent to utilize new knowledge and strategies on the job, and significant increases in knowledge of opioid impact on communities. Nearly all participants reported satisfaction with the sessions, and they felt that the sessions contributed to their overall understanding of strategies to support children impacted by the opioid crisis. Important to behavior change, participants reported they were utilizing new knowledge, skills, and strategies then planned to continue to use new practices in their work. Specifically, during the case discussions, evidence-based recommendations and strategies to improve outcomes of the child/family were shared so participants could implement immediately in their practice. These discussions are solution-focused and consider the resources and practices of each distinct state or region. By gaining access to a group of experts (SCOPE hub team), the participants engaged in complex conversations about the interactions of child and family characteristics, SDOH, and the systems within which they existed. They worked collaboratively, in real-time, to develop practical solutions that could then be implemented in their state/region. SCOPE, therefore, is a promising approach for increasing the capacity of providers to support children with NAS and their families, thereby increasing the capacity of our healthcare system to respond to this epidemic.

### Limitations

Although we had a large sample and found compelling evidence that SCOPE is an effective program that can rapidly reach a wide range of providers, there are several limitations to note. First, due to the nature of this project, we are limited in the conclusions that can be made. Specifically, this was a program evaluation of the impact a professional training program on providers, rather than a controlled study of patient outcomes. There is no control group or alternative condition with which to compare our results. Therefore, we cannot conclude that the changes reported here are unequivocally due to SCOPE. However, given that there are limited professional development opportunities related to NAS, and none have the same focus as SCOPE, it seems possible that this program was the primary driver of this effect. Future research would be needed to make a stronger claim and to explore more nuanced reasons why some participants might not have been satisfied with the program.

Further, the implementation of SCOPE was not monitored at each site. Therefore, it is not possible to claim that there was consistency in the professional development received by all trainees. However, the ability to adapt curricula to meet local needs is a key feature of ECHO. Indeed, after the initial training of the state sites, they were all free to customize the program to suit their local needs. This ability to adapt the program could be one reason we were able to reach such a large geographic region. It may also account for some of the knowledge gains we observed. That is, providers may have been more engaged in the material because each state team was able to make the content relevant. While we cannot confirm based on our data, this type of adaptation is a known element of successful implementation (44, 45). Regardless, it will be important to better understand the elements of this program that were critical drivers of the outcomes. Future research on SCOPE within an implementation science framework could clarify this more effectively.

Finally, given that this was a program evaluation, there were limitations in our ability to measure some outcomes directly. For instance, providers' implementation strategies were self-reported, the number of children with NAS on their workload was estimated and knowledge gains were indirectly assessed. Therefore, these findings should be interpreted with care. Nonetheless, given the large scale of this program, this is encouraging evidence that SCOPE contributed to the desired outcomes.

## Conclusion

As illustrated in this project, the ECHO model is feasible, acceptable, and effective, and offers a timely approach to increase the capacity of the states and communities to meet emerging health needs, such as that of the opioid crisis. When paired with a specifically designed curriculum and a nationwide method for dissemination, it can be a powerful tool for (1) enabling equitable access, (2) building a diverse and skilled workforce, (3) improving and innovating through evaluation, research, and quality improvement, and (4) building and maintaining a strong organizational infrastructure for public health. Decision-makers and administrators are called to support and extend the use of interprofessional education and training modalities, such as ECHO, to improve family outcomes. Our findings should be used to advocate for systems-level funding to support interprofessional training and capacity building across all systems inclusive of partners across sectors.

Specific to the opioid crisis, Project SCOPE was a highly effective method of increasing the capacity of interdisciplinary providers to address cross-system issues impacting children and families. This approach provides a training modality which allowed interdisciplinary professionals to obtain continuing education on a range of developmental, social-emotional, health, and systems-level topics arming them with skills to effectively support families and children impacted by substance use disorders and related trauma. Moreover, this model can be rapidly adapted and deployed to reach a wide geographic region, especially areas that are highly affected by the opioid crisis and underserved rural communities. Further, state teams who have participated in SCOPE have the benefit of an ongoing infrastructure and local community of practice to minimize the impact of the opioid epidemic on children and families in the future. These children are increasingly noted to have physical health impacts, behavioral health concerns, and educational needs. SCOPE can effectively offer interdisciplinary professionals from cross-sector perspectives the necessary knowledge, skills, and opportunities for collaboration to prevent and/or mitigate long term sequalae of prenatal opioid exposure on children and families, while the ECHO model can serve as a promising practice for public health prevention for a range of complex health care needs or public health emergencies.

## Data Availability

The raw data supporting the conclusions of this article will be made available by the authors, without undue reservation.

## Footnotes

^1^Neonatal Opioid Withdrawal Syndrome (NOWS) has been the term used to describe specific prenatal opioid exposure in infants (47); however, we will use the more inclusive term NAS throughout this paper.
